# The prevalence of psychiatric disorders among visitors to faith healers in Saudi Arabia

**DOI:** 10.12669/pjms.305.5434

**Published:** 2014

**Authors:** Fahad D. Alosaimi, Youssef Alshehri, Ibrahim Alfraih, Ayedh Alghamdi, Saleh Aldahash, Haifa Alkhuzayem, Haneen Albeeeshi

**Affiliations:** 1 Fahad D. Alosaimi, MD, Assistant Professor, King Saud University, Riyadh, Saudi Arabia.; 2 Youssef Alshehri MD, Senior Registrar, Prince Sultan Medical Military City, Riyadh, Saudi Arabia.; 3 Ibrahim Alfraih, Psychiatry Resident, Saudi Commission for Health Specialties, Riyadh, Saudi Arabia.; 4 Ayedh Alghamdi, Psychiatry Resident, Saudi Commission for Health Specialties, Riyadh, Saudi Arabia.; 5 Saleh Aldahash, Psychiatry Resident, Saudi Commission for Health Specialties, Riyadh, Saudi Arabia.; 6 Haifa Alkhuzayem, Psychiatry Resident, Saudi Commission for Health Specialties, Riyadh, Saudi Arabia.; 7 Haneen Albeeeshi, Psychiatry Resident, Saudi Commission for Health Specialties, Riyadh, Saudi Arabia.

**Keywords:** Faith Healing, Spiritual Therapies, Mental Disorders

## Abstract

***Objective:*** We investigated the prevalence of psychiatric disorders among visitors to Faith Healers (FHs) in Riyadh, Saudi Arabia. We also studied the sociodemographic profiles for these visitors, in addition to their past psychiatric history, reason(s) for seeking FH help, and past and current treatment experience with FHs.

***Methods:*** We conducted a cross-sectional study among the visitors (n=321) to a number of faith healing settings in Riyadh, Saudi Arabia using a specially designed questionnaire and validated Arabic version of The Mini International Neuropsychiatric Interview.

***Results:*** Most of the participants were young adults (35.1±10.8 years) and males with intermediate and secondary levels of education who had not sought medical help prior to their visits. A high proportion of the FH visitors have diagnosable mental illnesses. Depressive and anxiety disorders were the most prevalent among the study participants; few visitors were affected by psychotic or bipolar disorders.

***Conclusions:*** The present study provides insight for understanding the type of patients with psychiatric disorders who visit Faith Healers.(FHs). The study highlights the tendency of psychiatric patients in Saudi Arabia to visit FHs, which could reflect the importance of further studies to clarify the impact of FHs on the management of those patients.

## INTRODUCTION

Psychiatric disorders are considered among the 20 most disabling conditions worldwide. One of the most common psychiatric disorders is unipolar major depression, which is projected to be the first leading cause of disease burden in 2030.^1^ Although the success rates for the treatment of many common psychiatric disorders are equal or exceed the success rates for many other medical disorders,^2^ accessing psychiatric services across the globe is still limited by a severe stigma that deters patients with psychiatric illnesses from seeking professional help. For example, only 41% of adult Americans who had a psychiatric disorder in 2012 received mental health services.^3^ ^{Formatting Citation}^In developing countries, the majority of patients with psychiatric disorders counsult traditional healers before seeking medical help.^4^

In Saudi Arabia, where the Islamic religon is influential, suprenatural forces such as witchcraft, evil eye, and spirits are culturally accepted.^5^^,^^6^ Furthermore, the traditional healing practices performed by religious Faith Healers (FHs) are primarily spiritual and faith-based. To name a few, these practices include reading specific verses from the Holy Quran and sayings by Prophet Mohammad (peace be upon him).^7^ In spite of the advances in the mental health services in Saudi Arabia,^8^ a considerable number of patients with psychiatric illnesses still consult FHs before seeing mental health professionals.^9^

Several previous studies conducted in Saudi Arabia focused on the attitudes and practice of FHs.^7^^,^^10^ One community study targeted families living together in a household in Riyadh and investigated the sociodemographic characteristics of complementary and alternative medicine visitors.^11^ Two additional studies addressed traditional healing experiences using a psychiatric outpatient clinic sample.^9^^,^^12^ To our knowledge, there have been no previous studies conducted in Saudi Arabia that have studied the characteristics of visitors to FHs within faith healing settings.

Therefore, our study investigated the prevalence of psychiatric disorders among FHs visitors in Riyadh, Saudi Arabia. The visitors’ sociodemographic profiles were studied, in addition to their past psychiatric history, reasons for seeking FH help, and past and current treatment experience with FHs. We hypothesize that a large number of FH visitors have a diagnosable mental illness and that a large number of mentally ill patients are receiving inadequate psychiatric care and possibly harsh treatments in faith healing settings.

## METHODS

***Population:*** The current study was conducted among visitors to faith healing settings in Riyadh, Saudi Arabia. A faith healing setting was defined as a specific place designated to run structured faith healing practices. The study includes all individuals 18 years and older, both adult males and females. Participants were required to give written consent to participate in the study.

***Study design:*** A cross-sectional study was carried out between September 2012 and July 2013. The study obtained all required ethical approval from the institutional review board at the Faculty of Medicine at King Saud University, Riyadh, Saudi Arabia.

***Questionnaire:*** We administered a specially designed questionnaire (designed by the study authors) and a validated Arabic version of The Mini International Neuropsychiatric Interview (M.I.N.I. 6.0) to all participants.

The M.I.N.I. is a short, structured diagnostic interview that has been validated against the much longer Structured Clinical Interview for DSM diagnoses (SCID-P) in English and French and against the Composite International Diagnostic Interview for ICD-10 (CIDI) in English, French, and Arabic.^13^^,^^14^ The study questionnaire included sociodemographic characteristics of the study participants: age, gender, marital status, education, occupation, and monthly income. The questionnaire assessed the type of past medical and psychiatric disorders, the way the psychiatric disorders were diagnosed and treated, and the patients' beliefs regarding the etiology of their psychiatric disorders. The questionnaire also assessed the reason for the current visit to the FH, whether medical help sought, and the types of faith healing interventions used for the current complaint. A multi-disciplinary committee covering psychiatry, faith healing, and epidemiology validated the content of the questionnaire. The questionnaire was then piloted on a small number of participants (n=20) before being widely distributed. The wording and suggested answers were modified for some questions based on the feedback from the pilot sample.

***Recruitment:*** Given the lack of the data about the prevalence of psychiatric disorders in the Saudi community, our sample size was estimated according to the sample size used in a similar study conducted in Sudan.^15^ A total of 400 questionnaires were distributed by the authors of our study to available patients in a faith healing setting, i.e., convenience sampling. Four out of six FHs allowed us to run the study in their places. One FH refused to cooperate with us because he thought we were trying to recruit his patients to our own psychiatric clinics. Another FH refused to participate without giving any reason. The participation rate was 80% of all contacted patients (321/400).

***Statistical Analysis:*** The data were analyzed using SPSS Pc+ version 21.0 (Chicago, IL, USA) statistical software. Descriptive statistics (mean, standard deviation (SD), and percentages) were used to describe the quantitative, categorical, and outcome variables. Pearson’s chi-squared test was used to examine the association between the categorical outcome variables. A p value of <0.05 and 95% confidence intervals were used to report the statistical significance and precision of the estimates.

## RESULTS

***Sociodemographic characteristics (*** Table-I ***): ***The mean age of the 321 study subjects visiting FHs was 35 years, with a higher proportion of males (83.5%) than females (16.5%). There were more married subjects (62.4%) than subjects who were single (30.4%) or divorced or widowed (7.2%). Subjects with intermediate and secondary educational levels were more numerous (63.6%) than subjects with other levels of education. The subjects with monthly incomes less than 10,000 Saudi Riyals (SR) (2,667$US) constituted 71.7% of the sample; 63.3% of individuals were working.

**Table-I:** Distribution of sociodemographic variables of study subjects visiting FHs

**Variables**	**No. (%)**	**p value**
Age (years) Mean(SD) = 35.1(10.8)		
Gender (n=321)		<0.0001
Male	268(83.5)	
Female	53(16.5)	
Marital Status (n=319)		<0.0001
Single	97(30.4)	
Married	199(62.4)	
Divorced or Widowed	23(7.2)	
Educational level (n=319)		<0.0001
Illiterate and Primary	43(13.5)	
Intermediate and Secondary	203(63.6)	
University and Diploma	67(21.0)	
Masters and Ph.D.	6(1.9)	
Occupation (n=316)		<0.0001
Student	30(9.5)	
Working	200(63.3)	
Not working	86(27.2)	
Monthly Income (n=230)		<0.0001
<10,000 SR (2,667 $US)	165(71.7)	
≥10,000 SR	65(28.3)	

***Past psychiatric history (*** Table-II ***): ***The prevalence of self-reported past psychiatric illness and past medical illness was found to be 23.4% and 40.2%, respectively. Among those who reported having a psychiatric illness, depression was highly prevalent (34.7%), as were schizophrenia (14.7%) and other illnesses (18.7%). A non-negligible fraction of individuals did not know what was their diagnosis was (17.3%). The vast majority of individuals (97.4%) received their psychiatric diagnosis from a physician. Approximately 21.3% of individuals had received anti-depressant medications for their psychiatric illness.

Participants reported that the practices of FHs to treat them were composed of reading the Holy Quran (95.6%), using blessed water (84.7%), and blessed olive oil (60.1%). The water and olive oil were blessed by having the FH recite the Holy Quran on them. Sadly, about 4.4% of individuals had been exposed to beating, choking, or electrical shocks as treatment interventions.

The participants attributed their psychiatric illnesses to: magic (5.5%), evil eye (12.3%), and social and financial stress (16.5%). About 49.3% reported more than one of these reasons. However, none of the individuals attributed his psychiatric illness to biological reasons.

**Table-II:** Prevalence of past psychiatric history of study subjects who visit FHs and past illness related variables

**Past illness and its variables**	**No. (%)**
Past psychiatric illness	75(23.4)
Past medical illness	129(40.2)
***Past psychiatric illness***	
Depression	26(34.7)
Schizophrenia	11(14.7)
Bipolar disorder	6(8.0)
Depression and anxiety	5(6.7)
Others	14(18.7)
Unknown to the participant	13(17.3)
***Diagnosis of psychiatric illness made by***	
Physician	73(97.4)
Others	2(2.6)
***Psychiatric medication***	
Anti-depressants	16(21.3)
Anti-psychotic	7(9.3)
Benzodiazepine	6(8.0)
Unknown	32(42.7)
More than one	5(6.7)
None	7(9.4)
***Past treatment interventions by FHs (n=183)*** *	
Reading Quran	175(95.6)
Blessed water	155(84.7)
Blessed olive oil	110(60.1)
Honey	78(42.6)
Seder (Rhamnus)	72(39.3)
Other herbs	13(7.1)
Beating, choking, and electrical shocks	8(4.4)
Others	13(7.1)
***Self-reported reasons for mental illness***	
Magic	4(5.5)
Evil eye	9(12.3)
Social and financial stress	12(16.5)
More than one	36(49.3)
Don’t know	14(18.7)

* The subjects have used more than one method.

***The current visits related variables (*** Table-III ***): ***About 74.1% of the subjects were visiting the FH for the treatment of their medical or psychiatric illness. The reported reasons for seeking treatment from FHs were: religious reasons, effectiveness, or family wishes. However, most of the participants (50.5%) thought of more than two reasons. The majority of individuals (65.5%) met the FH at Esteraha, which is a designated place for practicing faith healing.

Only 41.2% of individuals had sought medical help for their current problem, while 50.9% felt that they derived no benefit from medical treatment. Only 39.7% of individuals were treated by psychiatrists and 60.3% were treated in other medical specialties. About 78.1% of individuals were taking medications for their illnesses. Reading the Quran (95.9%) and using blessed water (71.3%) were the most sought-after types of traditional treatment for their disorders.

**Table-III:** Distribution of Variables of Study Subjects Related to Current Visits to FHs

**Variables**	**No. (%)**
***Reason for current visit***	
Treatment of medical or psychiatric illness	223(74.1)
Suspicion of witchcraft or evil eye	16(5.3)
Upon family member request	11(3.6)
Preventing future illnesses	19(6.3)
Others	42(13.9)
***Reason for seeking treatment from FHs***	
Religious reasons	72(23.9)
Perception of effectiveness	40(13.3)
Family wishes	27(9.0)
More than one	152(50.5)
Others	10(3.3)
***Places of meeting FHs (n=293)***	
Patient’s house	8(2.7)
FH’s house	12(4.1)
Esteraha	192(65.5)
More than one	81(27.6)
***History of seeking medical help for current problem***	
Yes	124(41.2)
No	177(58.8)
***Medical specialty where previous help sought (n=121)***	
Psychiatry	48(39.7)
Other medical specialties	73(60.3)
***Type of Medical treatment (n=114)***	
Medications	89(78.1)
Psychotherapy	8(7.0)
Others	17(14.9)
***Benefit of medical treatment (n=112)***	
Yes	55(49.1)
No	57(50.9)
***Current Treatment intervention from FHs (n=268)*** *	
Reading Quran	257(95.9)
Blessed water	191(71.3)
Blessed olive oil	0 (0)
Honey	99(36.9)
Seder (Rhamnus)	95(35.4)
Other herbs	4(1.5)
Beating, choking, and electrical shocks	0 (0)
Others	18(6.7)

* Used more than one method.

***Prevalence of lifetime/current psychiatric illnesses (*** Table-IV ***)*** *: *We assessed different psychiatric disorders among the study subjects using the M.I.N.I. instrument. Depressive disorders were most prevalent (34.9%), followed by anxiety disorders (18.7%). We found that 6.9% of the subjects had psychotic disorders and 5% had bipolar disorders. Other disorders, including alcohol, substance-related, and eating disorders were prevalent in 12.8% of subjects who were visiting FHs.

**Table-IV:** Prevalence of different lifetime/current psychiatric illnesses assessed by the M.I.N.I. among the subjects visiting FHs (n=321).

**Type of disorder**	**No.(%)**	**95% confidence interval**
Psychotic disorders	22(6.9)	(4.4–10.2)
Bipolar disorders	16(5,0)	(2.9–8.0)
Depressive disorders	112(34.9)	(29.7–40.4)
Anxiety disorders	60(18.7)	(14.6–23.4)
Other disorders	41(12.8)	(9.3–16.9)

***The association between the lifetime/current psychiatric illnesses diagnosed by M.I.N.I. instruments and sociodemographic variables*** ***(*** Table-V ***): ***Among the sociodemographic characteristics of the subjects visiting FHs, gender, marital status, and occupation were statistically significantly associated with the psychotic disorders. Male subjects (8.2%) were statistically significantly more likely than females to be affected by psychotic disorders. Single individuals (13.4%) and people who were divorced or widowed (13.0%) were also more likely to have psychotic disorders than married subjects (3%); this result is statistically significant. Unemployed subjects had a higher rate of psychotic disorders than students (3.3%) and working subjects (4%); this finding was statistically significant. Other variables such as age and monthly income were not significantly correlated with the presence of psychotic disorders.

Bipolar disorders were prevalent in young adult (31.46 years); the data show a statistically significant difference in the mean age of subjects who had bipolar disorders and those who did not. Moreover, there was a statistically significant difference among subjects (21.7%) who got divorced or were widowed and who had bipolar disorders compared with single (4.1%) and married (3.5%) subjects. Other variables such as gender, occupation, and monthly income were not statistically significantly associated with bipolar disorders (Table-V).

Monthly income is the only variable that has a statistically significant association with the presence of depressive and anxiety disorders. Subjects with monthly incomes less than 10,000 SR had a higher incidence of depressive and anxiety disorders, 39.9% and 25.5%, respectively, compared with individuals who had monthly incomes of more than 10,000 SR (28.4% and 12.2%, respectively).

**Table-V:** Association among psychotic, bipolar, depressive, anxiety disorders and sociodemographic variables of study subjects visiting FHs

***Study variables***	***Psychotic disorders No.(%)***			***Bipolar disorders No.(%)***			***Depressive disorders No.(%)***			***Anxiety disorders No.(%)***		
	***Yes***	***No***	***p value***	***Yes***	***No***	***p value***	***Yes***	***No***	***p value***	***Yes***	***No***	***p-value***
Age (in years)* Mean (SD)	31.9(7.7)	35.3(10.9)	0.07	31.4(6.8)	35.3(10.9)	0.04	36.1(10.1)	34.6(11.1)	0.22	33.1(9.4)	35.5(11)	0.08
Gender												
Male	22(8.2)	246(91.8)	0.03	11(4.1)	257(95.9)	0.10	90(33.6)	178(66.4)	0.27	49(18.3)	219(81.7)	0.67
Female	0	53(100)		5(9.4)	48(90.6)		22(41.5)	31(58.5)		11(20.8)	42(79.2)	
Marital Status												
Single	13(13.4)	84(86.6)	0.002	4(4.1)	93(95.9)	0.001	25(25.8)	72(74.2)	0.06	20(20.6)	77(79.4)	0.41
Married	6(3.0)	193(97)		7(3.5)	192(96.5)		77(38.7)	122(61.3)		37(18.6)	162(81.4)	
Divorced or widowed	3(13.0)	20(87)		5(21.7)	18(78.3)		10(43.5)	13(56.5)		2(8.7)	21(91.3)	
Occupation												
Student	1(3.3)	29(96.7)	0.002	0(0)	30(100)	0.18	8(26.7)	22(73.3)	0.54	10(33.3)	20(66.7)	0.05
Working	8(4.0)	192(96.0)		9(4.5)	191(95.5)		74(37)	126(63)		74(37)	126(63)	
Not working	13(15.1)	73(84.9)		7(8.1)	79(91.9)		30(34.9)	56(65.1)		12(14)	74(86)	
*Monthly Income*												
<10,000SR	8(5.2)	145(94.8)	0.16	7(4.6)	146(95.4)	0.95	61(39.9)	92(60.1)	0.04	39(25.5)	114(74.5)	0.003
≥10,000 SR	14(9.4)	134(91.6)		7(4.7)	141(95.3)		42(28.4)	106(71.6)		18(12.2)	130(87.8)	

## DISCUSSION

Our study showed that high proportions of FH visitors have diagnosable psychiatric illnesses. This result is in contrast to a small proportion of participants who self-reported having a past psychiatric history. More than half of the participants had not sought medical help for their complaints. Of those who had sought help, half of them did not derive any benefit from the medical treatment. Depression and anxiety disorders were the most prevalent diseases among the study participants in comparison to psychotic or bipolar disorders. Individuals with psychotic disorders were more likely to be unemployed and people with psychotic or bipolar disorders were more likely to be unmarried. Most of the participants attributed their illness to more than one reason, including social and financial stress and other faith-based explanations such as magic and evil eye, which are common beliefs in Saudi Arabia.

Moreover, our study highlights the importance of integrating adequate psycho-education into routine clinical practice. The high percentage of patients who received psychiatric or medical care without perceived benefits may be due to the frustration of these individuals stemming from the chronicity of their illness. Having a more adequate explanation about the course and prognosis of their illnesses might have increased their satisfaction of the routine clinical care they received.

Most of the visitors to the FHs were young adults with intermediate and secondary levels of education. This fact indicates that people with higher educational levels may seek medical help instead of a faith healing treatment. FH treatments are similar to what has been reported in other studies – recitations of the Holy Quran and the use of blessed water and olive oil. Beating, choking, and electrical shocks have been used with a small number of visitors to the FHs; such harsh treatments have been reported in the literature.^16^

In one household survey done in Riyadh city and its suburbs by Al-Rowais et al.^11^ the investigators examined the reasons and health problems associated with seeking help from traditional healers. The traditional healers in this study were not restricted to those who practiced a faith-based treatment but also included other alternative therapeutic methods such as herbal treatments that lie beyond traditional medicine. Approximately 42% of study subjects consulted traditional healers sometime in their life. Similar to our finding, there was an inverse relationship between educational level and the likelihood of visiting a traditional healer. The most commonly used treatment was Holy Quran recitations (62.5%). However, in contrast to our study, the elderly and females were more likely to visit traditional healers than younger persons.

More than one study has investigated the sociodemographic and clinical characteristics of FH visitors among psychiatric outpatients in Saudi Arabia.^9^^,^^12^ One study showed that a high proportion of individuals had visited traditional healers before seeking psychiatric treatment,^9     ^which was similar to our findings. Both these studies showed that women used traditional medicine more often. Surprisingly, both also showed that educational level and employment did not seem to be correlated with visiting traditional healers. One investigation showed that the diagnosis of schizophrenia was significantly associated with visiting traditional healers^9^ while the other study revealed no difference among the psychiatric diagnoses.^12^

Similar studies have been conducted in countries with similar faith backgrounds as Saudi Arabia. One study from Pakistan investigated the prevalence, classification, and treatment of psychiatric disorders among visitors to FHs^17^ and found that the most prevalent diagnosis is major depressive episodes followed by generalized anxiety disorder, which is consistent with our findings. Another study conducted in Sudan found that psychotic disorders and bipolar disorders were the most prevalent among visitors to traditional healing centers. However, in these centers, the FHs practice in inpatient settings and help seekers were kept in their facilities.^15^ In our study, the setting was an outpatient setting where the visitors comes to see the FHs but do not stay there, which is inconvenient for individuals with severe mental illnesses such as psychotic disorders or bipolar disorder.

Our study has a number of limitations, including the lack of data related to the prevalence of psychiatric disorders in the Saudi community; such information would be helpful for estimating more accurately the sample size and help in interpreting the study results. Also, the use of convenience sampling may not accurately represent the population under study; in our sample we had a higher proportion of male participants than female participants, which may be due to the reluctance of females to participate in such a study as well as the restriction imposed by some faith healers against interviewing females by male data collectors.

In conclusion, the present study provides insights for understanding the characteristics of the visitors to FHs and the psychiatric disorders they suffer from. The study highlights the tendency of psychiatric patients in Saudi Arabia to visit FHs, which could reflect the importance of further studies to clarify the impact of FHs on the management of those patients. We advocate educating FHs in identifying common psychiatric symptoms and referring patients in need of psychiatric care. Overall, this study calls for active involvement of non-governmental organizations, universities and media in public education, reducing psychiatric stigma, and bridging the treatment gap in mental health.

## Notes

***Source of funding:*** None.
